# Role of PatS and cell type on the heterocyst spacing pattern in a filamentous branching cyanobacterium

**DOI:** 10.1093/femsle/fnx154

**Published:** 2017-07-21

**Authors:** Laura A. Antonaru, Dennis J. Nürnberg

**Affiliations:** Department of Life Sciences, Imperial College, London SW7 2AZ, UK

**Keywords:** multicellularity, cyanobacteria, Stigonematales, cell differentiation, nitrogen fixation, heterocyst spacing pattern, PatS

## Abstract

Cell differentiation is one of the marks of multicellular organisms. Terminally specialised nitrogen-fixing cells, termed heterocysts, evolved in filamentous cyanobacteria more than 2 Gya. The development of their spacing pattern has been thoroughly investigated in model organisms such as *Anabaena sp.* PCC 7120. This paper focuses on the more complex, branching cyanobacterium *Mastigocladus laminosus* (Stigonematales). Contrary to what has been previously published, a heterocyst spacing pattern is present in *M. laminosus* but it varies with the age of the culture and the morphology of the cells. Heterocysts in young, narrow trichomes were more widely spaced (∼14.8 cells) than those in old, wide trichomes (∼9.4 cells). Biochemical and transgenic experiments reveal that the heterocyst spacing pattern is affected by the heterocyst inhibitor PatS. Addition of the pentapeptide RGSGR (PatS-5) to the growth medium and overexpression of *patS* from *Anabaena* sp. PCC 7120 in *M. laminosus* resulted in the loss of heterocyst differentiation under nitrogen deprivation. Bioinformatics investigations indicated that putative PatS sequences within cyanobacteria are highly diverse, and fall into two main clades. Both are present in most branching cyanobacteria. Despite its more complex, branching phenotype, *M. laminosus* appears to use a PatS-based pathway for heterocyst differentiation, a property shared by *Anabaena/Nostoc*.

## Teaser

In the filamentous branching cyanobacterium *Mastigocladus laminosus*, the heterocyst spacing pattern is regular but cell-type specific, and depends on PatS, the protein that controls cell differentiation.

## INTRODUCTION

The cyanobacteria are one of the oldest and morphologically most diverse groups of bacteria, ranging from unicellular forms to filamentous, branching species that show various degrees of cell differentiation (Rippka *et al.*^1979^). Under nitrogen depletion, some filamentous cyanobacteria differentiate photosynthetically active vegetative cells into specialised nitrogen-fixing cells called heterocysts. Heterocysts protect the highly oxygen-sensitive nitrogenase from oxygen by degrading their (oxygen-evolving) photosystem II, forming additional layers in their cell wall (envelope) to slow O_2_ diffusion into the cell and increasing their respiration rate to keep the O_2_ concentration low (Kumar, Mella-Herrera and Golden 2010). However, this comes at the cost of being dependent on neighbouring vegetative cells for carbon skeletons and for most of their energy. Consequently, cyanobacteria form heterocysts at a certain frequency. Cyanobacteria that form heterocysts are classified in Sections IV (Nostocales) and V (Stigonematales), depending on their ability to divide in a single plane or in several planes, leading to the formation of true branches. In Section IV cyanobacteria belonging to the genera *Anabaena* and *Nostoc*, heterocysts are formed in non-random, semi-regular intervals of 10 to 20 cells along the filament (Wilcox, Mitchison and Smith 1973). Most studies have focussed on the strain *Anabaena* sp. PCC 7120 as model organism, but also related strains such as *Anabaena variabilis* ATCC 29413 and *Nostoc punctiforme* PCC 73102 (ATCC 29133) have been investigated (Meeks and Elhai 2002).

Many genes play a role in establishing the pattern. Under nitrogen deprivation, the transcription factors NtcA and HetR activate and repress the transcription of hundreds of heterocyst differentiation genes in a hierarchical manner including *patS* and *hetN* (Herrero, Picossi and Flores 2013).

Both proteins, PatS and HetN, contain the pentapeptide, RGSGR (also known as PatS-5), which has been shown to inhibit heterocyst differentiation when added to the growth medium in *Anabaena* sp. PCC 7120 and *N. punctiforme* ATCC 29133 by interacting with HetR and abolishing its DNA-binding capacity (Yoon and Golden 1998; Huang, Dong and Zhao 2004; Feldmann *et al.*^2011^; Risser, Wong and Meeks 2012; Hu *et al.*^2015^). In *Anabaena* sp. PCC 7120, PatS is a short peptide of 17 amino acids that is processed in the producing cells to the pentapeptide PatS-5 or the hexapeptide PatS-6 (ERGSGR) from the octapeptide PatS-8 (CDERGSGR), which is then transferred to neighbouring cells (Wu *et al.*^2004^; Corrales-Guerrero *et al.*^2013^; Zhang *et al.*^2017^). Inactivation of *patS* in *Anabaena* sp. PCC 7120 results in the formation of heterocysts in the presence of combined nitrogen and in the formation of multiple contiguous heterocysts (MCH) under nitrogen deprivation (Yoon and Golden 1998), whereas overexpression of the gene inhibits heterocyst differentiation (Wu *et al.*^2004^). While *patS* is expressed early after nitrogen step down in small cell clusters, *hetN* expression follows later in the heterocysts (Callahan and Buikema 2001). Deletion of *hetN* results in the formation of MCH under nitrogen depletion (Black and Wolk 1994; Callahan and Buikema 2001). However, it has been recently suggested that *hetN* is not enough to inhibit heterocyst formation at a late stage but additional products of nitrogen fixation must assist (Muñoz-García and Ares 2016). At least in *A. variabilis* ATCC 29413, it appears that exogenously supplied nitrogen has a more important effect on patterning than endogenous fixed nitrogen (Thiel and Pratte 2001).

Although our understanding of pattern formation in Section IV cyanobacteria has increased significantly over the last years, almost nothing is known about pattern formation in more complex cyanobacteria such as the Section V cyanobacterium *Mastigocladus laminosus*. *Mastigocladus laminosus* forms a dense cellular network of intertwined trichomes of cells with different morphology, ranging from narrow and cylindrical (1.5–2.0 μm in diameter) to wide and rounded (8–11 μm) (Balkwill, Nierzwicki-Bauer and Stevens 1984; Kaštovský and Johansen 2008). Cells can differentiate into heterocysts, hormogonia (motile filaments), akinetes (resting cells) and necridia (releasing cells) (Schwabe 1960). Its importance as a nitrogen fixer in hot springs around the world is well documented (Fogg 1951; Finsinger *et al.*^2008^; Soe *et al.*^2011^; Mackenzie, Pedro and Diez 2013; Alcamán *et al.*^2015^; Hutchins and Miller 2016) with a high heterocyst frequency of up to 28.3% in wide filaments (Stevens, Nierzwicki-Bauer and Balkwill 1985). However, a regular heterocyst spacing pattern was not observed (Nierzwicki-Bauer, Balkwill and Stevens 1984).

Here, we show that *M. laminosus* SAG 4.84 has a regular heterocyst spacing pattern that varies depending on the age of the culture and the morphology of the cells. Heterocysts in young, narrow filaments were more widely spaced than those in old, wide filaments. Addition of the pentapeptide RGSGR to the growth medium and expression of *patS* from *Anabaena* sp. PCC 7120 in *M. laminosus* inhibited heterocyst differentiation under nitrogen deprivation, suggesting a similar mechanism of pattern regulation in cyanobacteria of Section IV and V. Bioinformatics analysis on the distribution of PatS-like sequences revealed the presence of two clades.

## MATERIALS AND METHODS

### Determination of heterocyst spacing pattern

*Mastigocladus laminosus* SAG 4.84 was grown in liquid Castenholz medium with added nitrate (Castenholz D (Castenholz 1988)) at 45°C under constant white light illumination of 30 μmol photons m^−2^ s^−1^ and shaking (120 rpm). The culture was used to inoculate nitrate-free media (Castenholz ND (Castenholz 1988)) and induce heterocyst formation. As the cyanobacterium grows in macroscopic clumps, which are typical for this species (Muster *et al.*^1983^), the culture was homogenised by several passages through a needle (diameter 1.2 mm) with a syringe prior to inoculation. The culture was washed four times with Castenholz ND medium using centrifugation (5 min at 3220 × g) to completely remove nitrate.

**Figure 1.**
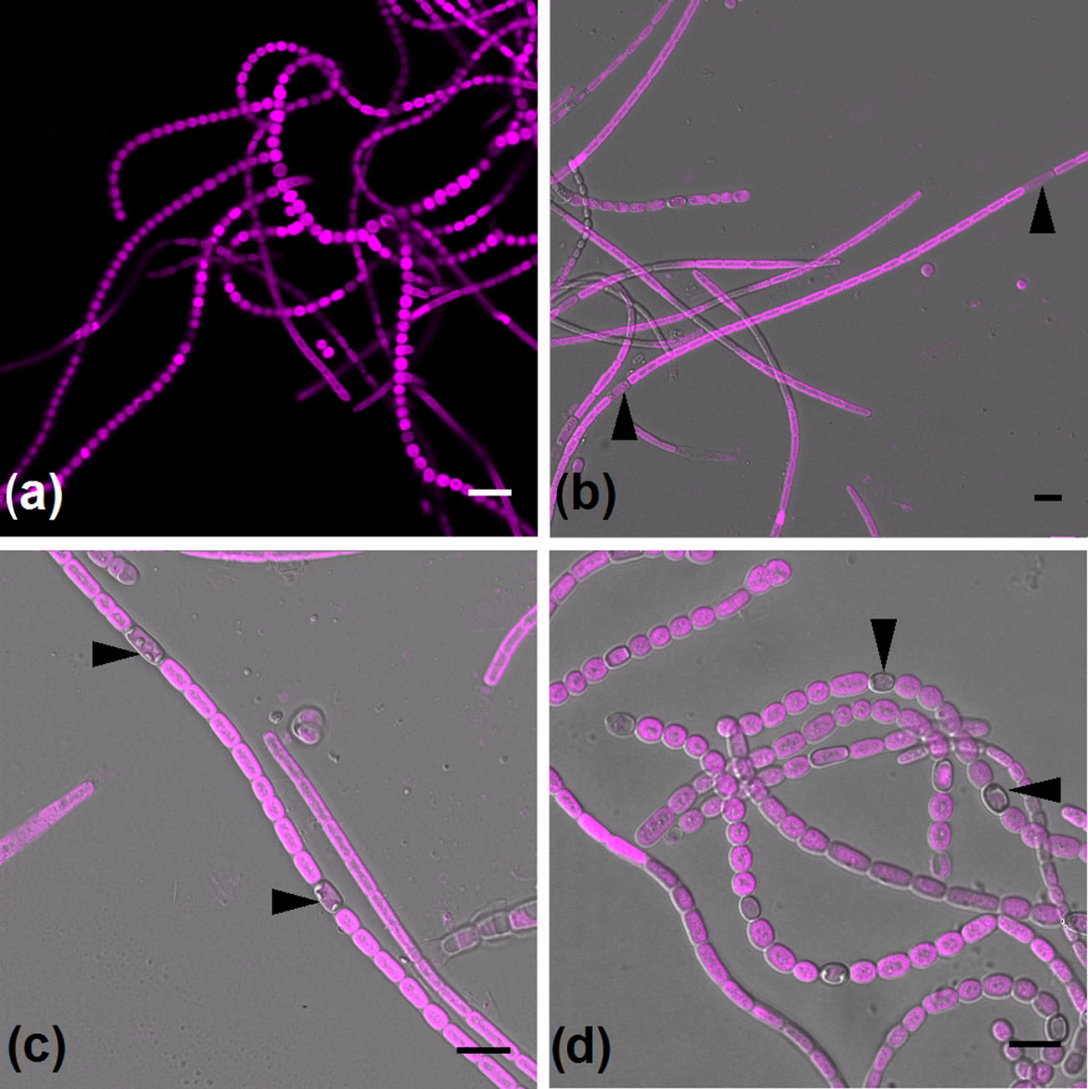
Morphology of *M. laminosus* trichomes. (**a**) Cellular network of cells of various morphology. As cells senesce, their phenotype changes from narrow, squared-end cylinders (**b**) to intermediate ellipsoidal forms (**c**) and eventually wide spheroids and spheres (**d**). Heterocysts are indicated by arrows. Images show an overlay of the bright field view (gray) and chlorophyll fluorescence (magenta, 488 nm excitation, 670–720 nm emission). Micrographs were taken 14 days (a, b, c) and 3 months (d) after nitrogen step down. Note the reduced fluorescence in heterocysts. Scale bars, 10 μm.

For determination of the heterocysts spacing pattern, a Kyowa Medilux-12 light microscope with a ×100 oil-immersion objective was used. The number of cells between two heterocysts was counted, and noted down alongside the morphology of the cells according to Nürnberg *et al.* (2014). If the counting spanned branches, the shortest distances were considered. Cells were grouped into (i) ‘narrow’, (ii) ‘ellipsoidal’ and (iii) ‘round’ (Fig. 1). The time points chosen for counting were at 13–16 days post-inoculation, and at 21–22 days. Counting heterocyst spacing patterns took place regularly over several days, in an attempt to collect enough data for each cell type. Earlier time points (2–5 days) were characterised by heterocysts too uncommon to facilitate large-scale counting.

To verify the results, a stationary phase culture was used to inoculate new Castenholz ND media. Growth conditions were the same as stated above. Measurements were repeated at 5–8 days and at 13–14 days post-inoculation.

For visualisation, images were taken with a Zeiss LSM-510 inverted confocal microscope using 488 nm excitation and a 670–720 nm emission detection range for chlorophyll (autofluorescence). Images were analysed with ImageJ 1.48 software (Schneider, Rasband and Eliceiri 2012).

### Effect of extraneous PatS pentapeptide RGSGR on cell growth

*Mastigocladus laminosus* and *Anabaena* sp. PCC 7120 grown in nitrate-containing media were washed four times with nitrate-free medium (Castenholz ND for *M. laminosus*, BG11_0_ for *Anabaena*) (Castenholz 1988) and used to inoculate 7.5 ml media on six-well plates. *Mastigocladus laminosus* samples were homogenised prior inoculation with a 1.2-mm diameter syringe needle. Two micromolar of the PatS RGSGR pentapeptide (kindly provided by Anna-Winona Struck) were added and plates were incubated at 30°C, 120 rpm and 30 μmol photons m^−2^ s^−1^. Direct visual and microscopic observations by light microscopy were performed 6 and 14 days after inoculation as described above.

### Generation of cyanobacterial mutants and phenotypic analysis

The *patS* gene from *Anabaena* sp. PCC 7120 (*asl2301*) was cloned into the self-replicating plasmid pRL25C (Wolk *et al.*^1988^) using BamHI. First, the *patS* gene was amplified through PCR using Q5 High-Fidelity DNA Polymerase and primers (FD: TCAAACATGAGAATTATGAAGGCAATTATGTTAGTG, REV: TCGTCTTCAAGAATTCTATCTACCACTACCGCG). Then, the PCR products were cloned into pRL25C using In-fusion cloning technique (Clontech). The resulting plasmid was named pLA4 and the presence of the gene was verified by sequencing.

pLA4 was transferred to *M. laminosus* by conjugation as described by Elhai and Wolk (1988), generating strain ICLA4. Plasmid pRL25C was used as control. Two *Escherichia coli* strains were used: HB101 containing the helper plasmid pRL623 and the donor plasmid (pRL25C or pLA4) and ED8654 containing the conjugative plasmid pRL443 (Elhai *et al.*^1997^). The *E. coli* cultures were mixed with *M. laminosus* cells corresponding to 15 μg chlorophyll *a* per plate, as measured by spectrophotometry according to Mackinney (1941). Cells were then spread on filter membranes on 1% (w/v) agar Castenholz D plates supplemented with 5% (v/v) LB medium, and incubated for 3 h in the dark at 30°C before exposing to normal light conditions. After 24 h of incubation, the membrane was transferred to a 1% (w/v) agar Castenholz D plate, and for selection every 48 h to 72 h transferred to an agar plate supplemented with 30 μg ml^−1^ neomycin, an antibiotic concentration that has been proven useful for selection in the closely related strain *Fischerella muscicola* PCC 7414 (Stucken *et al.*^2012^). Once resistant colonies were visible on the filter membranes, eight of these were selected from each strain and re-streaked twice on Castenholz D agar plates with 30 μg ml^−1^ neomycin. For growth analysis on solid media, each strain was re-streaked on agar plates with and without addition of nitrate and incubated at 30°C and 20 μmol photons m^−2^ s^−1^. For liquid cultures, one colony was picked from each agar plate and grown in 7.5 ml of Castenholz D media supplemented with 30 μg ml^−1^ neomycin, in six-well plates. Once colonies reached a diameter of ∼5mm, the samples were washed four times with Castenholz ND medium and grown in six-well plates as described above. The heterocyst spacing pattern was assessed after 12 days as previously stated.

### Bioinformatics analysis

The presence of patterning genes in heterocyst-forming cyanobacteria was investigated in 7 Section IV strains and 14 Section V strains the genomes of which have been assembled at least to the contig level. For HetR and HetN, the *Anabaena* sp. PCC 7120 amino acid sequences (WP_010996495.1 and P37694.2) were used to search the protein sequence data (cut-off e-90). For PatS and its alternative, the RGSGR sequence was used as a query. Short sequences (<150 aa) of unknown function were stored for further use, and phylogenetic analysis was later used to confirm homology. For *Nostoc* sp. NIES-3756 and *Anabaena variabilis* ATCC 29413, only nucleotide *pats* sequences were available. Translations were employed.

A multiple alignment was performed using Clustal Omega algorithms (10 iterations), with Seaview 4.6 software (Gouy, Guindon and Gascuel 2010). Five additional PatS homologues were added to the dataset (see Table S2 for accession numbers). A maximum-likelihood phylogeny was built from it (Seaview 4.6), highlighting two distinct clades and motifs that extended beyond hexapeptides. They were termed ‘classical’ and ‘alternative’ PatS. Phylogeny settings: aLRT branch support, empirical amino acid equilibrium frequencies, optimised invariable sites, tree searching best of NNI and SPR, five random starts, tree topology optimised. The tree was annotated with iTOL (Letunic and Bork 2016). The stability of the clades was tested with different alignments (T-Coffee) and tree-building methods (Bayesian, neighbour joining).

The motifs were further investigated with MEME, a motif-finding webserver (Bailey and Elkan 1994). Settings: default for classical PatS; one occurrence per sequence, two motifs, maximum width 20, for the alternative PatS. The increased stringency was prompted by high similarity and putative motif paralogy.

## RESULTS

### Cell-type specific heterocyst spacing pattern

To test for the presence of a heterocyst spacing pattern in *Mastigocladus laminosus*, cells were transferred from nitrate-containing to nitrate-free media. *Mastigocladus laminosus* has a complex life cycle, where initially narrow cells become round. A stationary culture shows mainly round cells and was used to inoculate nitrate-free media, thus not only inducing heterocyst development but also the formation of branches and of narrow trichomes.

**Figure 2.**
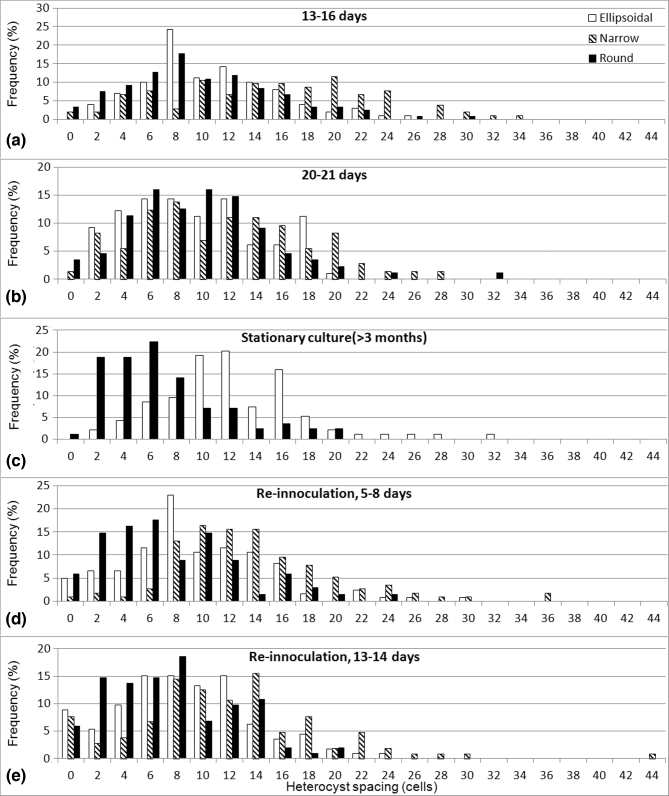
Heterocyst spacing pattern in *M. laminosus* at different time points after nitrogen deprivation: (**a**) 13–16 days, (**b**) 20–21 days and (**c**) more than 3 months (stationary culture). Cells from a stationary culture were then used to inoculate new nitrogen-free media and heterocyst spacing patterns were determined after 5–8 days (**d**) and 13–14 days (**e**). Cells were grouped in narrow, ellipsoidal and round. Y-axis represents percentage out of all filaments counted for the given time range. A total of 321 filaments were counted for (a); 259 for (b); 179 for (c); 306 for (d); 318 for (e).

Only few heterocysts were observed shortly after inoculation (2–5 days). These were particularly present in old trichomes formed of round cells; no heterocysts were observed in narrow filaments. At a later stage, 13–16 days after inoculation, mature heterocysts were frequently observed in both narrow and wide trichomes. Counting of heterocysts revealed the presence of a pattern that varies between cell types. The heterocysts in narrow filaments were more widely spaced (∼14.8 cells apart) than those in filaments with round cells (∼9.4 cells). An intermediate state was also defined with cells that appear ‘ellipsoidal’. The spacing for ellipsoidal cells was with ∼10.8 cells between two heterocysts similar to that found for round cells (Fig. 2a, Table S1). The variance was significantly higher in narrow trichomes (7.5 cells) than in trichomes with ellipsoidal (5.2 cells) and round cells (5.7 cells) and the spacing in narrow filaments varied from 0 (double heterocyst) to 34 cells between two heterocysts.

The wide-spacing effect disappeared at 21–22 days, with heterocysts separated by 9.7 ± 5.6 cells on average (Fig. 2b, Table S1). In addition, at this time, fewer narrow filaments were observed, suggesting a potential slowdown of growth. As narrow filaments are the first to form (Thurston and Ingram 1971; Nierzwicki *et al.*^1982^; Nürnberg *et al.*^2014^), maintaining an equivalent rate of growth overall would imply that the rate of change from narrow to wide trichomes changed as well.

No narrow trichomes were observed in the stationary phase culture (3 months; Fig. 2c, Table S1). However, whereas previously the patterning in ellipsoidal- and round-cell trichomes was not statistically different, in this sample the number of cells between ‘round’ heterocysts was significantly reduced to 6.5 ± 4.6 cells. This fits with observations that in stationary cultures, all cells acquire the round phenotype, and heterocysts are more closely spaced together.

Upon re-inoculation (5–8 days), a similar effect to the first inoculation step was observed (Fig. 2d, Table S1). Branching occurred and heterocysts in narrow trichomes were separated by a higher number of cells (∼13.4 cells) than those in trichomes with ellipsoidal cells (∼9.2) and round cells (∼7.1). However, within each of the trichome types, spacing between heterocysts was significantly reduced in the second culture, being more typical of the ‘stationary phase’ (Fig. 2e, Table S1). This could be explained by the fact that the cells had already acclimatised to nitrate-free media.

### Effect of PatS on heterocyst development

**Figure 3.**
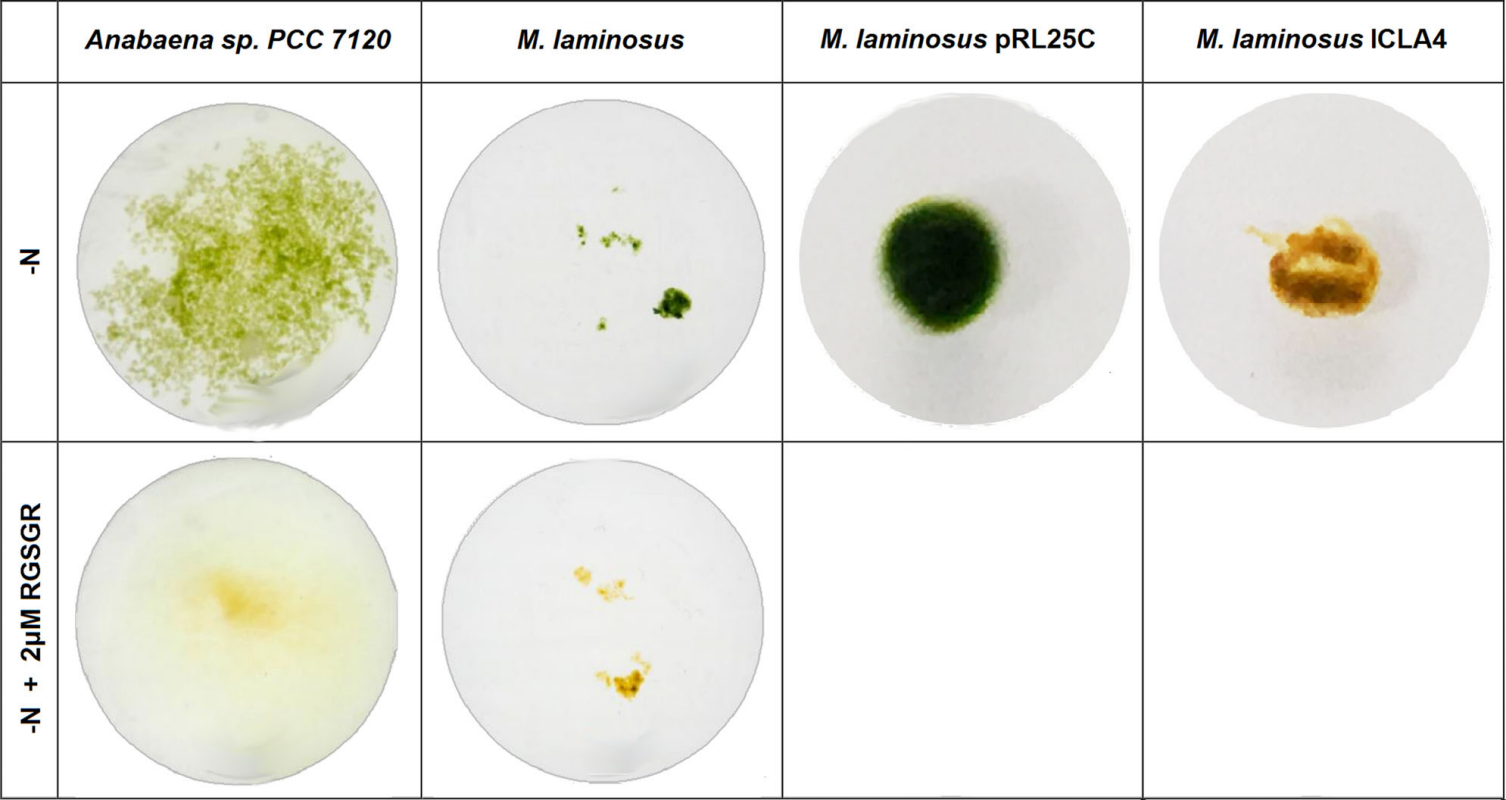
Effect of pentapeptide RGSGR and PatS on growth of *Anabaena* sp. PCC 7120 and *M. laminosus* under nitrogen deprivation. Addition of RGSGR to the growth medium (second row) leads to cell death and bleaching. Expression of *patS* in *M. lamnosis* (ICLA4) shows a similar effect when grown in nitrate-free media (last column). The control strain with pRL25C was able to grow under nitrogen depletion condition. Photographs were taken 12 days after inoculation.

In cyanobacterium *Anabaena* sp. PCC 7120, PatS is a key protein in establishing the semiregular heterocyst spacing pattern (Yoon and Golden 1998). We tested whether this is also the case for *M. laminosus* by adding the pentapeptide RGSGR and expressing the *patS* gene from *Anabaena* sp. PCC 7120 in *M. laminosus*. Addition of RGSGR at a concentration of 2 μM inhibited heterocyst formation in both *Anabaena* sp. PCC 7120 and *M. laminosus*. Six days after inoculation, the differences between experimental and control cultures were evident, with the RGSGR cultures being largely bleached (Fig. 3). No heterocysts were observed under the microscope. Cultures that were grown in nitrate-free media without RGSGR remained green and showed heterocysts.

In a second approach, the 54-nucleotide *patS* gene from the *Anabaena sp.* PCC 7120 genome was cloned into two versions of the self-replicating plasmid pRL25C (Wolk *et al.*^1988^), resulting in plasmid pLA4. As a control plasmid, pRL25C without any insert was used. The plasmids were introduced through conjugation into *M. laminosus* using methods established for *Anabaena* (Elhai and Wolk 1988) and *Nostoc* strains (Flores and Wolk 1985; Cohen *et al.*^1994^) and recently also for Section V cyanobacteria (Stucken *et al.*^2012^; Zhao *et al.*^2015^). The colonies obtained were restreaked twice on Castenholz D media and then transferred to nitrate-free and nitrate-containing agar plates. No growth was observed on nitrate-free plates for strains expressing *patS* from *Anabaena* sp. PCC 7120 (Fig. 3). Only very few heterocysts but many dead cells were observed under the microscope in transgenic cultures, in contrast with the control strain. The growth-inhibitory effect was not seen on N+ plates. In liquid media, the majority of the *M. laminosus* cells with the *patS*-containing plasmid died following nitrogen starvation (Fig. 3). Microscopy revealed that they contained only very few scattered heterocysts. In comparison, the control had a large number of heterocysts with a semiregular spacing pattern.

### Distribution of RGSGR-containing proteins among Section V cyanobacteria

**Figure 4.**
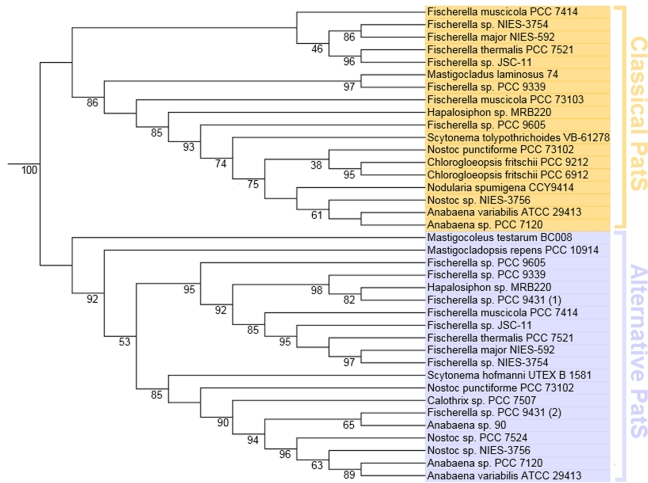
Maximum-likelihood phylogenetic tree of PatS and putative homologues. Orange labels mark amino acid sequences that cluster together with the PatS from the model organism *Anabaena* sp. PCC 7120 (‘classical PatS’). Blue labels mark alternative sequences with proline-rich motifs. There is a clear split between the two groups (branch support 1). The tree was automatically rooted. Branch support values (aLRT) > 0.6 are listed.

Mutagenesis studies indicated that the pentapeptide RGSGR is the main functional part of PatS and HetN (Yoon and Golden 1998; Higa *et al.*^2012^; Corrales-Guerrero *et al.*^2013^). We performed a Blast search to test how widely distributed RGSGR-containing proteins are among Section V cyanobacteria. HetN homologues were found by similarity with the *Anabaena sp.* PCC 7120 sequence, although only a subset of them have an RGSGR motif and all of them belong to Section IV cyanobacteria (Table 1) (Corrales-Guerrero *et al.*^2014^). Similar to *Anabaena* sp. PCC 7120, most cyanobacteria of Section V contain HetN-like sequences, PatS and an additional sequence that is similar to PatS, which we term ‘alternative PatS’ (Table 1, Fig. 4). These short RGSGR sequences of unknown function clustered together with PatS, but the split between the classical PatS and the alternative PatS was marked and distinct from other RGSGR-containing sequences like HetN homologues. The separation was evident irrespective of the alignment method (Clustal Omega, T-Coffee), tree-building algorithm (Bayesian, neighbour-joining, maximum likelihood) or data editing (removing insertions that only occur in one organism).

**Table 1.** Distribution of RGSGR-containing heterocyst-patterning genes in Section IV and V cyanobacteria.

			‘Classic’	‘Alternative’
Strain name	*hetR*	*hetN*	*patS*	*patS*
***Section IV***				
*Anabaena* sp. 90	•	–	–	•
*Anabaena* sp. PCC 7120	•	•^*^	•	•
*Anabaena variabilis* ATCC 29413	•	•^*^	–	•
*Calothrix* sp. PCC 7507	•	–	–	•
*Nodularia spumigena* CCY9414	•	–	•	•
*Nostoc* sp. NIES-3756	•	•^*^	–	•
*Nostoc* sp. PCC 7524	•	•^*^	–	•
*Nostoc punctiforme* PCC 73102	•	•	•	•
***Section V***				
*Chlorogloeopsis fritschii* PCC 6912	•	•	•	–
*Chlorogloeopsis fritschii* PCC 9212	•	•	•	–
*Fischerella major* NIES-592	•	•	•	•
*Fischerella muscicola* PCC 7414	•	•	•	•
*Fischerella* sp. JSC-11	•	•	•	•
*Fischerella* sp. NIES-3754	•	•	•	•
*Fischerella* sp. PCC 9339	•	•	•	•
*Fischerella* sp. PCC 9431	•	–	–	•
*Fischerella* sp. PCC 9605	•	•	•	•
*Fischerella muscicola* PCC 73103	•	•	•	•
*Fischerella thermalis* PCC 7521	•	•	•	•
*Hapalosiphon* sp. MRB220	•	•	•	•
*Mastigocladopsis repens*	•	•	–	•
*Mastigocladus laminosus* 74	–	•	•	–
*Mastigocladus laminosus* UU774	•	•	–	–
*Mastigocoleus testarum* BC008	•	•	–	•
*Scytonema hofmanni* UTEX B 1581	•	–	–	•
*Scytonema tolypothrichoides* VB-61278	•	–	•	•

The presence (•) or absence (-) of *hetN*, the classic *patS* and the alternative *patS* are indicated. Only strains with completed genome sequences were considered for Section IV in addition to the model strains *A. variabilis* ATCC 29413 and *N. punctiforme* PCC 73102. All genomes of Section V cyanobacteria that are assembled to the contig level were considered. As an indicator for nitrogen fixation, the master regulator gene *hetR* was included. *hetN* genes that encode the RGSGR motif are marked with an asterisk.

**Figure 5.**
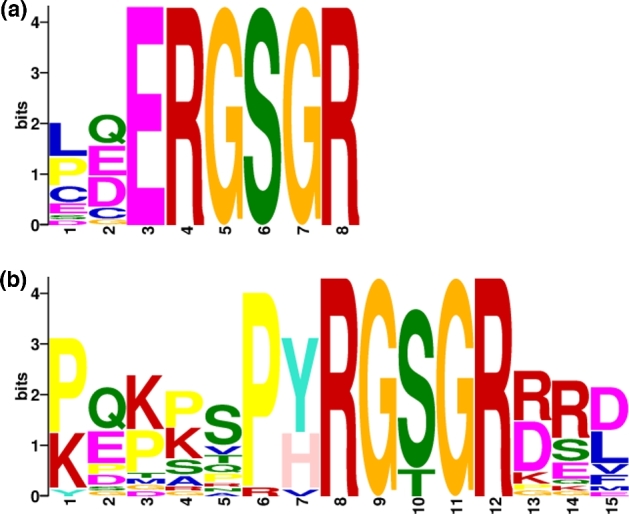
Conserved motifs in putative PatS homologues as identified by MEME. ERGSGR sequences (**a**) are associated with the classic PatS while a more complex RGSGR motif is present in alternative PatS sequences (**b**). Note that in alternative PatS sequences show a proline-rich sequence preceding the main motif.

Further analysis of the sequences revealed that in contrast to the 17-aa long PatS sequence in *Anabaena* sp. PCC 7120, most putative PatS and alternative PatS sequences (90% of 38) were between 65 and 115 amino acids in length, and motif analyses using MEME (Bailey and Elkan 1994) revealed the presence of an extended ERGSGR motif in putative PatS sequences while alternative PatS sequences were characterised by multiple conserved prolines preceding a Y/HRGSGR motif (Fig. 5).

## DISCUSSION

The formation of special nitrogen-fixing cells in cyanobacteria is one of the most important examples of prokaryotic cell differentiation. Its simplicity of having only two cell types, photosynthetically active vegetative cells and nitrogen-fixing heterocysts lead to *Anabaena* and *Nostoc* species becoming model organisms (Meeks and Elhai 2002; Herrero, Stavans and Flores 2016). However, cyanobacteria include species which are more complex: those which divide in a secondary plane and those which differentiate into hormogonia, necridia and/or akinetes. *Mastigocladus laminosus* is one of these organisms. A previous study suggested that nitrogen starvation stimulates extensive heterocyst differentiation with heterocyst frequencies of 28.3% in wide trichomes and 17.5% in narrow trichomes only 44 h after nitrogen step down. However, no obvious spacing pattern was observed, leading to the hypothesis that the organism lacks the precise control process needed to regulate heterocyst spacing that is found in *Anabaena* spp. (Nierzwicki-Bauer, Balkwill and Stevens 1984; Stevens, Nierzwicki-Bauer and Balkwill 1985). In our study, *M. laminosus* shows a clear heterocyst spacing pattern after nitrogen step down. However, it is only formed after several days in nitrate-free media when using a long-term/stationary culture (>6 months). At the early stage, only few heterocysts could be seen in wide trichomes but extensive branching with the formation of narrow trichomes was observed. After 13–16 days nitrogen step down, heterocysts were present in both narrow and wide trichomes and a spacing pattern could be determined. On the other hand, wide trichomes with round cells and ellipsoidal cells showed that two heterocysts were separated by 9.4 and 10.8 vegetative cells, respectively. In narrow trichomes, the average distance between heterocysts was with 14.8 cells significantly higher and varied from 0 (double heterocysts) to more than 30 vegetative cells. We attribute the wide spacing in young, narrow trichomes to the very high cell division rate and the high molecular flux between cells that was demonstrated in an earlier study by FRAP (fluorescence recovery after photobleaching) experiments (Nürnberg *et al.*^2014^). In narrow trichomes, inhibitors such as PatS-5 might be transmitted too quickly to establish the stable concentration gradient that is required for the formation of heterocysts at the position of the lowest inhibitor concentration. The transfer of molecules in cyanobacteria appears to occur via septal junctions, which are protein complexes composed of SepJ, FraC, FraD and other as yet unidentified proteins (Bauer *et al.*^1995^; Flores *et al.*^2007^; Nayar *et al.*^2007^; Merino-Puerto *et al.*^2010^, ^2011^; Nürnberg *et al.*^2015^). It has been suggested that different complexes exist that have different selectivity for molecules (Mullineaux and Nürnberg 2014). The importance of these complexes for molecular transfer in *M. laminosus* or other Section V cyanobacteria remains to be investigated.

The RGSGR pentapeptide (PatS-5) has been previously shown to inhibit heterocyst formation in *Anabaena* PCC 7120 (Yoon and Golden 1998) and *Nostoc punctiforme* ATCC 29133 (Risser, Wong and Meeks 2012). This is also the case for *M. laminosus*. However, purely biochemical experiments should be taken with caution. Previously, a complementation experiment of a *ΔpatS* mutant in *Anabaena* sp. PCC 7120 showed that, while the integration of a gene encoding the RGSGR-containing octapeptide (PatS-8) into the bacterial chromosome produced a phenotype that was indistinguishable from the wild type, the insertion of just the pentapeptide RGSGR could not restore the normal heterocyst spacing pattern (Corrales-Guerrero *et al.*^2013^). A recent study confirmed that PatS-8 is processed to the shorter PatS-5 and PatS-6, which then might diffuse along the filament (Zhang *et al.*^2017^). Expression of the full-length *patS* from *Anabaena* sp. PCC 7120 in *M. laminosus* showed an effect similar to the addition of RGSGR to the growth medium. The strain was unable to form heterocysts and to survive under nitrogen depletion.

In addition, it has been shown that in *N. punctiforme* ATCC 29133 an unequal distribution of the pattern-related receptor protein PatN along the filament can predate nitrogen starvation, and influence which cells are likely to differentiate (Risser**, Wong and Meeks 2012). It would be interesting to know how this relates to the different cell types in *M. laminosus.*

Although the genome sequence of *M. laminosus* SAG 4.84 is currently unknown, the 16S rRNA sequence and the gene sequence of the septal protein SepJ (also known as FraG) suggested high similarity to the sequenced strain *Fischerella muscicola* PCC 7414 (Nürnberg *et al.*^2014^). We performed a Blast search and confirmed that similar to *Anabaena* sp. PCC 7120 most cyanobacteria of Section V contain HetN-like sequences and PatS but also an ‘alternative PatS’. Such alternative forms have been previously found in other cyanobacteria such as *Cylindrospermopsis raciborskii* CS-505 (Stucken *et al.*^2010^) and *Anabaena* sp. 90 (Wang *et al.*^2012^) (both Section IV), and they appear to be present in most heterocyst-forming cyanobacteria (Jeffrey Elhai, personal communication). Zhang, Chen and Zhang (2009) furthermore reported that some filamentous but non-heterocystous strains such as *Arthrospira platensis* (Section III) possess an ‘alternative PatS’, which despite being 90 aa long, is able to function as a heterocyst inhibitor in *Anabaena* sp. PCC 7120. Similar to these results, our data set suggests that the short form of PatS is less abundant and most sequences were between 65 and 115 amino acids in length. This has important implications on intercellular transport dynamics and the processing of the peptide as only small molecules are assumed to diffuse between cells via septal junctions or a continuous periplasm (Mullineaux and Nürnberg 2014; Nieves-Morión, Mullineaux and Flores 2017). The exact route and molecule remains to be determined.

Particularities of sequence differences between clades might relate to function. A high number of conserved proline residues were detected in alternative PatS sequences. In general, proline-rich repeats have been suggested to have a ‘sticky arm’ effect, binding reversibly to other proteins (Williamson 1994). It is notable that the septal protein SepJ also contains a proline-rich linker region in many heterocyst-forming cyanobacteria (Flores *et al.*^2007^; Mariscal *et al.*^2011^).The function of these alternative PatS proteins remains to be explored in further studies.

## SUPPLEMENTARY DATA

Supplementary data are available at *FEMSLE* online.

## Supplementary Material

Supplemental material Supplementary data are available at *FEMSLE* online.
