# Supplementary material for: Role of PatS and cell type on the heterocyst spacing pattern in a filamentous branching cyanobacterium
Source: FEMS Microbiol Lett. 2017 Jul 21;364(15):fnx154. doi: 10.1093/femsle/fnx154 (PMC5812504; doi:10.1093/femsle/fnx154)
Supplement: Supplemental material — Supplementary data are available at FEMSLE online. [file fnx154_supp.docx]

|  | **Narrow** | **Ellipsoidal** | **Round** |
| --- | --- | --- | --- |
| **13-16 days** | 14.8 ± 7.5 | 10.2 ± 5.2 | 9.4 ± 5.7 |
| **20-21 days** | 11.2 ± 6.4 | 9.0 ± 4.9 | 9.1 ± 5.4 |
| **Stationary culture** | - | 11.7 ± 5.4 | 6.5 ± 4.6 |
| **Re-inoculate, 5-8 days** | 13.4 ± 6.1 | 9.2±5.5 | 7.1 ± 5.2 |
| **Re-inoculate, 13-14 days** | 11.4 ± 7.2 | 8.3 ± 5.1 | 7.0 ± 4.6 |
| **Table S1.** Number of vegetative cells (mean and standard deviation) between heterocysts corresponding to different trichome types at various time-points. Dots and lines highlight whether the two table cells that they connect have statistically different values (red) or not (green). Blue represents borderline statistical significance, α = 0.05. Two-way ANOVA, Holm-Sidak method to correct for multiple comparisons. | | | |

| **Strain name** | **HetR** | **HetN** | **‘Classic’ PatS** | **‘Alternative’ PatS** |
| --- | --- | --- | --- | --- |
| ***Section IV*** | | | | |
| *Anabaena sp.* 90 | WP_015078258.1 | - | - | WP_015078262.1 |
| *Anabaena* *sp.* PCC 7120 | P27709.3 | P37694.2 | O52748.1 | BAB74031.1 |
| *Anabaena variabilis* ATCC 29413 | Q9F5Y3.3 , AAG24634.2 | ABA22211.1 | CP000117.1  (nucleotide)  157286-157233 | ABA19776.1 |
| *Calothrix sp.* PCC 7507 | WP_015131714.1 | - | - | WP_015131722.1 |
| *Nodularia spumigena* CCY9414 | AHJ28017.1 | - | AHJ31088.1 | WP_071839281.1^*^ |
| *Nostoc* *sp.* NIES-3756 | WP_067769022.1 | WP_067766726.1 | AP017295.1  (nucleotide)  1276254-1276307 | WP_067769009.1 |
| *Nostoc* *sp.* PCC 7524 | WP_015140333.1 | WP_015141496.1 | - | WP_015140326.1 |
| *Nostoc punctiforme* PCC 73102 | WP_012408409.1 | WP_012409766.1 | ACC83668.1 | WP_041565664.1 |
| ***Section V*** | | | | |
| *Chlorogloeopsis fritschii* PCC 6912 | WP_016873360.1 | WP_016877961.1 | WP_016878033.1 | - |
| *Chlorogloeopsis fritschii* PCC 9212 | WP_016873360.1 | WP_016877961.1 | WP_016878033.1 | - |
| *Fischerella major* NIES-592 | WP_016871868.1 | WP_073556599.1 | WP_073556052.1 | WP_062246735.1 |
| *Fischerella muscicola* PCC 7414 | WP_016868103.1 | WP_016870042.1 | WP_016868851.1 | WP_016870487.1 |
| *Fischerella sp.* JSC-11 | WP_009453740.1 | WP_009756823.1 | WP_009454941.1 | WP_009453748.1 |
| *Fischerella sp.* NIES-3754 | WP_016871868.1 | WP_062248016.1 | WP_062247157.1 | WP_062246735.1 |
| *Fischerella sp*. PCC 9339 | WP_017311371.1 | WP_017313163.1 | WP_026082084.1 | WP_017313078.1 |
| *Fischerella sp*. PCC 9431 | WP_026722965.1 | - | - | WP_026719406.1, WP_026723828.1 |
| *Fischerella sp*. PCC 9605 | WP_026732380.1 | WP_051470324.1 | WP_026734037.1 | WP_026732389.1 |
| *Fischerella muscicola* PCC 73103 | WP_016863540.1 | WP_016861857.1 | WP_016860387.1 | WP_016859831.1^*^ |
| *Fischerella thermalis* PCC 7521 | WP_016871868.1 | WP_009756823.1 | WP_009454941.1 | WP_009453748.1 |
| *Hapalosiphon* *sp*. MRB220 | WP_053458377.1 | WP_053455428.1 | WP_053457853.1 | WP_026719406.1 |
| *Mastigocladopsis repens* PCC 10914 | WP_017318187.1 | WP_017317848.1 | - | WP_017318179.1 |
| *Mastigocladus laminosus* 74 | - | WP_044448738.1 | WP_026082084.1 | - |
| *Mastigocladus laminosus* UU774 | KIY12088.1 (partial) | WP_044448738.1 | - | - |
| *Mastigocoleus testarum* BC008 | WP_027841706.1 | WP_027843613.1 | - | WP_058183641.1 |
| *Scytonema hofmanni* UTEX B 1581 | WP_029633330.1 | - | - | WP_029634580.1 |
| *Scytonema tolypothrichoides* VB-61278 | WP_048866876.1 | - | WP_048871236.1 | WP_048868074.1^*^ |
| **Table S2.** **Accession numbers for putative RGSGR-containing heterocyst-patterning sequences in Section IV and V of cyanobacteria.** Where not specified, these represent protein sequences. Only strains with completed genome sequences were considered for Section IV cyanobacteria in addition to the model strains *A. variabilis* ATCC 29413 and *N. punctiforme* PCC 73102. Genomes of Section V cyanobacteria that are assembled to the contig level were considered. ‘-‘ signifies that the gene was not found. ^*^ - not used in the phylogeny. | | | | |
